# Guidance on Multiplicity Analysis in Single-Trial Assessments: A No-Solution Equation

**DOI:** 10.3390/jmahp14030046

**Published:** 2026-08-04

**Authors:** Mondher Toumi, Bruno Falissard, Asma Jouini, Pascal Auquier, Claude Dussart, Laurent Boyer

**Affiliations:** 1 CEReSS/UR3279—Health Services Research and Quality of Life Center, Aix-Marseille University, 13385 Marseille, France; pascal.auquier@univ-amu.fr (P.A.); laurent.boyer@univ-amu.fr (L.B.); 2 CESP, INSERM U1018, Université Paris-Saclay, 94800 Villejuif, France; bruno.falissard@gmail.com; 3 Clever Access, Tunis 1058, Tunisia; asma.jouini@clever-access.com; 4 UR 4129 P2S Parcours Santé Systémique, University Claude Bernard Lyon 1, 7-11 rue Guilllaume Paradin, 69008 Lyon, France; claude.dussart@chu-lyon.fr; 5 Pharmacie Centrale, Hospices Civils de Lyon, 57 Rue Francisque Darcieux, Saint-Genis-Laval, 69230 Lyon, France

The European Health Technology Assessment (EU HTA) guidance on Multiplicity of Hypothesis Testing (MHT) [1] resembles a no-solution algebra equation. The hypothetico-deductive method used in inferential statistics, which this guidance references, relies on conclusive reasoning. However, the guidance fails to provide a clear resolution, leaving the matter to individual Member States (MS) despite its critical impact on statistical precision—a key pillar of validity in clinical trials for comparative effectiveness assessments in Joint Clinical Assessments (JCAs) [2].

MHT in inferential statistics increases the likelihood of false-positive results (Type I errors) [3]. There are two main approaches for addressing this issue: (1) mathematical models such as the Bonferroni correction, which inflates sample size, and (2) hierarchical hypothesis testing, which does not [3,4]. While the guidance acknowledges the problem, it neither resolves nor mitigates it.

Key unresolved questions include:

- Should each PICO (Population, Intervention, Comparison, Outcome) be considered an independent predefined analysis, thereby exempting it from MHT? If so, it must be defined before results are available, which is currently not the case.
- Why should individual MS determine how to handle MHT, despite its status as a state-of-the-art statistical method? The Member State Coordination Group on Health Technology Assessment (HTACG) deviates from the EU HTA Regulation, which mandates adherence to the state-of-the-art rigorous methodology.

Moreover, the guidance states that “*for some MSs, the unplanned post hoc analysis is more important for the PICO than the planned analysis…/…in such cases, JCA report will document the p-value, and it will be marked as nominal*” [1]. This contradicts best scientific practices, where post hoc analysis *p*-values should not be reported since their statistical assumptions do not hold. Instead, such analyses should be classified as descriptive exploratory findings without statistical testing [5]. Requesting multiple PICOs during the scoping phase, once clinical trial results are available and submitted to the HTACG, means that any non-predefined PICO analysis qualifies as post hoc and should not be subjected to hypothesis testing. If testing is conducted, MHT adjustment must be applied, but *p*-values should not be reported to avoid misleading conclusions.

The guidance requires identifying whether post hoc analyses originate from authorities or sponsors [1]. However, given that the JCA subgroup is “decontextualized and judgment-free [6],” post hoc analyses should be treated equally, regardless of the requester. Identifying the originator introduces implicit judgment, contradicting this principle. This contradiction invites a critical question: is the JCA process genuinely decontextualized, or is this principle selectively applied?

Moreover, this guidance clearly violates the established principles of hypothetico-deductive reasoning and MHT by allowing assessors to arbitrarily select a single time point, preferably the last data cut, in multiple time point analyses. This introduces subjective judgment, which does not align with the EU HTA regulation’s aim for objectivity and judgment-free JCAs [6]. Furthermore, the appropriateness of the chosen data cut-off depends on the endpoint and may affect the accuracy of the results: while it may be reasonable for hazard ratios or median survival, it is not for the probability of survival (e.g., a time-dependent measure that requires a complete view of the survival curve) [7].

The guidance specifies that if a MS requested a specific subgroup analysis, the results must be reported and the Credibility of Effect Modification Analyses (ICEMAN) [8] criteria may be used to interpret and assess the results of the submitted subgroup analyses. However, the ICEMAN checklist has been criticized for not considering the MHT related risk and its subjective judgment rating on a Visual Analogic Scale [9]. Free judgment is violated again.

The introduction of additional post hoc analyses and the use of the ICEMAN tool for effect modifiers are useful for HTA appraisals but not for a decontextualized clinical assessment. However, their inclusion contradicts the EU HTA regulation, which mandates adherence to state-of-the-art methodology, while ICEMAN is instead a heuristic approach used to assess credibility rather than ensure statistical rigor.

The guidance fails to address how the multiplicity of hypothesis testing—both within a single PICO and across multiple PICOs—impacts the degree of certainty of the comparative effectiveness of an assessed intervention versus a reference. This is the primary goal of the JCA subgroup [2].

In conclusion, the guidance provides a detailed discussion of multiplicity but fails to align with contemporary well established statistical standards. The post hoc nature of PICOs defined after trial results renders them unsuitable for hypothesis testing; therefore, no statistical tests should be performed, and *p*-values should not be reported to be aligned with EU HTA Regulation.

The lack of a systematic literature review, transparency, and accountability prevented the guidance’s authors from identifying viable solutions to these critical methodological issues. It remains unclear whether the guidance considers the implications of MHT on statistical precision and certainty in comparative effectiveness assessments—the goal of JCA.

This ambiguity likely arises from diverging perspectives between HTA bodies—HAS (National French Health Authority) [10], which demands strict control over multiple hypotheses testing, and G-BA (The German Federal Joint Committee) which adopts a more flexible approach [11,12]. Differences stem from Fisher’s and Neyman-Pearson’s approaches to hypothesis testing. Fisher used it for inductive inference, where the *p*-value assesses data against the null hypothesis without considering alternatives. Conversely, Neyman and Pearson viewed hypothesis testing as “inductive behavior,” making decisions (accepting or rejecting an alternative hypothesis) based on error probabilities rather than evidence quantification [13,14,15].

The HTACG faces two choices: either exclude MHT from its guidance, failing to address a key issue, or include it without resolving its complexities.

Given the constraints of the EU HTA Regulation, which limits judgment and flexibility while mandating evidence for multiple PICOs [6] (up to 13 in recent simulations [16]), resolving the MHT issue within the current framework is infeasible. The HTACG should withdraw this guidance and leave the matter to national authorities.

## Data Availability

No new data were created or analyzed in this study. Data sharing is not applicable to this article.

## Abbreviations

The following abbreviations are used in this manuscript:

EU	European Union
EU HTA	European Health Technology Assessment
G-BA	The German Federal Joint Committee
HAS	National French Health Authority
HTA	Health Technology Assessment
HTACG	The Member State Coordination Group on Health Technology Assessment
HTD	Health Technology Developers
ICEMAN	Instrument to assess the Credibility of Effect Modification Analyses
JCA	Joint Clinical Assessment
JSC	Joint Scientific Consultation
MHT	Multiplicity of Hypothesis Testing
MS	Member States
PICO	Population, Intervention, Comparison, Outcome
